# Impact of patisiran on polyneuropathy of hereditary transthyretin amyloidosis in patients with a V122I or T60A variant: a phase IV multicenter study

**DOI:** 10.1080/07853890.2025.2537347

**Published:** 2025-09-20

**Authors:** Yessar Hussain, Saurabh Malhotra, Brett W. Sperry, Francy Shu, Karthikeyan Ananthasubramaniam, Urvi Desai, Ugochukwu Egolum, Joel Fernandez, Miriam Freimer, Elizabeth Mauricio, Dianna Quan, Roy Small, Kelley Capocelli, Steven Roblin, Shaun Bender, Patrick Y. Jay, Elena Yureneva, Ronald Zolty

**Affiliations:** a Austin Neuromuscular Center, Austin, TX, USA; b Division of Cardiology, Cook County Health and Rush Medical College, Chicago, IL, USA; c Department of Cardiovascular Medicine, Saint Luke’s Mid America Heart Institute, Kansas City, MO, USA; d Department of Neuroscience, Baylor University Medical Center/Baylor Scott and White Health, Dallas, TX, USA; e Department of Cardiology, Henry Ford West Bloomfield Hospital, West Bloomfield Township, MI, USA; f Department of Neurology, Atrium Health Wake Forest University, Charlotte, NC, USA; g Northeast Georgia Medical Center, Division of Cardiology, Georgia Heart Institute, Gainesville, GA, USA; h Department of Cardiovascular Services, Morsani College of Medicine, University of South Florida, Tampa, FL, USA; i Department of Neurology, Wexner Medical Center, Ohio State University, Columbus, OH, USA; j Department of Neurology, Mayo Clinic, Jacksonville, FL, USA; k Department of Neurology, University of Colorado Anschutz, Aurora, CO, USA; l Department of Cardiology, Lancaster General Health/Penn Medicine, Lancaster, PA, USA; m Alnylam Pharmaceuticals, Cambridge, MA, USA; n Department of Cardiology, University of Nebraska Medical Center, Omaha, NE, USA

**Keywords:** ATTRv amyloidosis, patisiran, polyneuropathy, RNA interference, treatment

## Abstract

**Background:**

This study assessed the effectiveness and safety of patisiran in patients with V122I/T60A variant transthyretin (ATTRv) amyloidosis with polyneuropathy. These variants have been under-represented in previous trials of gene-silencing agents.

**Methods:**

This was a multicenter, phase IV study conducted at 27 sites in the USA. Patients were ≥ 18 years, diagnosed with ATTRv amyloidosis with polyneuropathy and a documented V122I or T60A variant. Patisiran-treated patients were enrolled prospectively, ambispectively, and retrospectively. The primary endpoint was the proportion of patients with a stable or improved polyneuropathy disability (PND) score at 12 months *vs*. baseline. Safety was monitored throughout the trial.

**Results:**

Sixty-seven patients were enrolled, of whom 58 received ≥ 1 dose of patisiran. In the efficacy population, 42/45 (93.3%) patients demonstrated stable or improved PND scores from baseline to Month 12. Patients also showed stable or improved quality of life, health status, autonomic symptoms, and cardiac function *vs*. baseline. Adverse events occurred in 13/42 (31.0%) patients in the prospective and ambispective cohorts; most were mild or moderate. No deaths or cardiac hospitalizations were considered related to patisiran.

**Conclusions:**

Patisiran demonstrated a consistent positive effect across multiple endpoints in patients with V122I/T60A ATTRv amyloidosis, including polyneuropathy manifestations.

## Introduction

Variant transthyretin (ATTRv) amyloidosis, also known as hereditary transthyretin amyloidosis, is a rare, underdiagnosed, rapidly progressive, debilitating, and fatal disease caused by variants in the *TTR* gene [1–4]. Globally, over 120 pathogenic transthyretin (TTR) variants have been described [1], of which V122I (p.Val142Ile) and T60A (p.Thr80Ala) are among the most commonly reported in the USA [2,3]. V122I is found in 3–4% of African Americans [2,4], and increases the risk of atrial fibrillation, heart failure (HF) hospitalization, and death in carriers who live to advanced age [5].

Irrespective of TTR genotype, most patients with ATTRv amyloidosis develop a mixed phenotype of polyneuropathy and cardiomyopathy in addition to manifestations in other systems such as gastrointestinal disturbance and autonomic neuropathy [3,6]. Most patients who have the T60A or V122I variants present mainly with cardiomyopathy [7,8], but some patients also have polyneuropathy [6,9]. For example, the risk of polyneuropathy among V122I carriers of African descent in the UK Biobank was significantly increased compared with non-carriers (HR = 6.9; *p* = 9.5 × 10^−4^). These findings were independently replicated in the Penn Medicine Biobank and Million Veteran Program populations [6]. Furthermore, polyneuropathy in patients with the V122I variant can be severe, causing patients to become wheelchair bound, or the only manifestation of ATTRv amyloidosis [10].

Patisiran is an RNA interference (RNAi) therapeutic administered once every 3 weeks *via* intravenous infusion, which suppresses production of both variant and wild-type TTR in the liver [11]. Patisiran was approved for treatment of the polyneuropathy of ATTRv amyloidosis based on the phase III, placebo-controlled APOLLO study; results demonstrated that patisiran had the potential to halt or reverse the progression of polyneuropathy, and improve quality of life, in a cohort of patients representative of the ATTRv amyloidosis with polyneuropathy patient population [12]. However, of the patients who received patisiran in the multinational APOLLO study, only 1/148 (0.7%) had the V122I variant and 12/148 (8.1%) had the T60A variant [12]. In other major clinical trials of ATTRv amyloidosis with polyneuropathy, the representation of patients with either the V122I or T60A variants has been similarly low [13–16] or nil [16]. Patisiran is not approved for the treatment of the cardiomyopathy of ATTRv amyloidosis.

Here, we report the results of a phase IV study in patients with ATTRv amyloidosis with polyneuropathy and a documented V122I or T60A variant who were treated with patisiran in a real-world setting. This study evaluated the effectiveness and safety of patisiran across a range of disease manifestations in patients with V122I/T60A ATTRv amyloidosis, with the aim of determining the impact of treatment on the multisystem burden associated with this disease.

## Materials and methods

### Patients

This was a multicenter, phase IV study (NCT04201418) conducted at 27 sites in the USA. Eligible patients were ≥ 18 years of age, with a diagnosis of ATTRv amyloidosis with polyneuropathy and a documented V122I or T60A variant. Patients had a polyneuropathy disability (PND) score of I–IIIB, a New York Heart Association (NYHA) class of ≤ 3, a Karnofsky Performance Status score of ≥ 60%, and adequate liver and renal function. Patients treated with TTR stabilizers (tafamidis or diflunisal) prior to baseline were eligible to continue these treatments during the study. However, patients who had received treatment with an antisense oligonucleotide agent (e.g. inotersen) or any investigational agent in the 6 months prior to baseline were excluded. Vutrisiran was not approved at the time of the study. All patients provided written informed consent.

### Trial design

Patients were enrolled into one of three cohorts based on prior patisiran exposure: 1) the prospective cohort: patients naive to patisiran at study enrollment with the intention to initiate commercial patisiran therapy; 2) the ambispective cohort: patients currently on commercial patisiran therapy for < 12 months at study enrollment; and 3) the retrospective cohort: patients on commercial patisiran therapy for ≥ 12 months prior to study enrollment, regardless of current treatment status at enrollment. Prospective patients were followed for approximately 12 months after the initiation of treatment with patisiran (from baseline until study completion (i.e. 12-month visit)). Data for ambispective patients were retrospectively abstracted from medical records from baseline to study enrollment to align with routine care encounters (dependent on when the patient enrolled), and patients were prospectively followed for the remainder of the first year of patisiran therapy. For the retrospective patients, data were retrospectively abstracted from patients’ medical records, if available, for the first year of patisiran treatment (at baseline and approximately 3, 6, and 12 months after patisiran initiation). Further details of data collection are outlined in the supplementary appendix.

Intravenous patisiran was administered for 12 months according to body weight: patients weighing < 100 kg received 0.3 mg/kg every 3 weeks and patients weighing ≥ 100 kg received 30 mg. Premedication was administered in accordance with the approved patisiran label. The reporting of the study adhered to CONSORT guidelines.

### Outcomes and assessments

The primary endpoint assessed the impact of patisiran on ambulatory status, measured by the proportion of patients with a stable or improved PND score at 12 months relative to baseline (the first dose of patisiran). A PND score of I represents sensory disturbances with preserved walking capability; II, impaired walking capability but without a stick or crutches; IIIA, walking with help of one stick or crutch; and IIIB, walking with the help of two sticks or crutches.

Exploratory endpoints evaluated the effectiveness of patisiran (mean change from baseline, assessed at baseline and at Months 3, 6, and 12, unless stated below) on the following: quality of life assessed by the Norfolk Quality of Life – Diabetic Neuropathy (Norfolk QOL-DN) questionnaire (range −4 to136, with higher scores indicating worse quality of life [17]); HF-associated health status and quality of life assessed by the Kansas City Cardiomyopathy Questionnaire – Overall Summary (KCCQ-OS) (range 0–100, with lower scores indicating worse health status [18]); autonomic symptoms assessed by the Composite Autonomic Symptom Score-31 (COMPASS-31) [19] questionnaire (range 0–100, with higher scores indicating more autonomic symptoms); clinical assessment of symptoms resulting from HF assessed by NYHA class (ranging from Class 1 (no symptoms) to Class 4 (symptoms at rest)), assessed at baseline and at Month 12; and cardiac stress assessed by N-terminal prohormone of brain natriuretic peptide (NT-proBNP) (higher values indicate a greater level of cardiac stress). Exploratory endpoints were assessed according to standard of care and as such were not available for all patients. Further details on primary and exploratory endpoints are provided in the supplementary appendix.

Safety was monitored throughout the trial in the ambispective and prospective cohorts. Select safety events were classified according to the Medical Dictionary for Regulatory Activities (MedDRA) System Organ Class and Preferred Term.

### Statistical analyses

The study planned to enroll approximately 60 patients and a sample size was not determined for statistical inferences. Efficacy endpoints were analyzed in patients who had completed 12 months of follow-up after initiating patisiran and taken ≥ 1 dose of patisiran (efficacy population). For the primary endpoint, frequency and percentage of patients with a stable or improved PND score at Month 12 relative to baseline were summarized. Continuous endpoints were summarized by mean, standard error of the mean, median, and range. Categorical variables were summarized using frequencies and percentages. Safety was analyzed in patients who received at least one dose of patisiran (safety population) in the ambispective and prospective cohorts.

## Results

### Patient disposition

**Figure 1.**
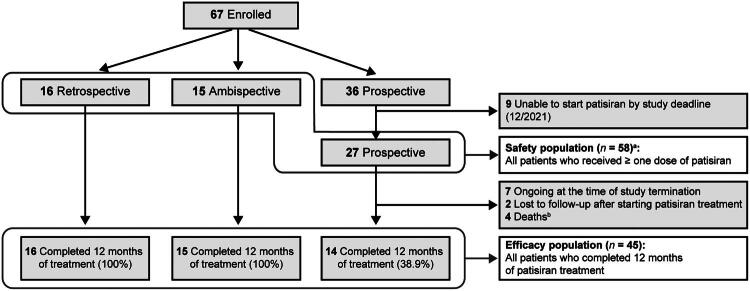
Patient disposition. ^a^Patients in the retrospective cohort were included in the safety population but excluded from the analysis of safety endpoints. ^b^Deaths unrelated to patisiran: one acute respiratory failure, one cardiac failure, one cardiogenic shock, and one unknown cause.

From December 2019 to June 2021, a total of 67 patients were enrolled in the study, of whom 58 received ≥ 1 dose of patisiran (safety population; Figure 1). Nine patients were unable to start patisiran by the study deadline.

Among 58 patients who received patisiran, 16, 15, and 27 were included in the retrospective, ambispective, and prospective cohorts, respectively. In the prospective cohort, seven patients were ongoing at the time of study termination, two patients were lost to follow-up, and four patients died. No patients in the ambispective or retrospective cohorts withdrew from the study. A total of 45 patients completed 12 months of follow-up assessment following initiation of patisiran treatment by study end date (Study period: December 18, 2019 to May 24, 2022) and were included in the efficacy analysis population. Safety findings were summarized for patients in the ambispective and prospective cohorts who received ≥ 1 dose of patisiran (*n* = 42).

### Baseline demographics and disease characteristics

The patient group treated with patisiran was mostly male (63.8%), with a mean (range) age of 65.1 (30–83) years (Table 1). The V122I variant was present in 45 (77.6%) patients, of whom 36 (80.0%) were Black or of African American ancestry and eight (17.8%) were White; the T60A variant was present in 13 (22.4%) patients, all of whom were White. Patients had experienced a mean delay of 2.7 years after initial symptom onset before being diagnosed with ATTRv amyloidosis (Table 1).

**Table 1.** Baseline demographics and disease characteristics (safety population, *n* = 58).

Characteristic	V122I variant (*n* = 45)	T60A variant (*n* = 13)	Total (*n* = 58)
Mean age^a^, mean (range), years	66.1 (33–83)	61.3 (30–78)	65.1 (30–83)
Male, *n* (%)	30 (66.7)	7 (53.8)	37 (63.8)
Race, *n* (%)			
Black	36 (80.0)	0	36 (62.1)
White	8 (17.8)	13 (100.0)	21 (36.2)
Not reported	1 (2.2)	0	1 (1.7)
Mean age at ATTRv amyloidosis symptom onset, mean (range), years	61.8 (25–82)	57.0 (18–77)	60.7 (18–82)
Mean age at ATTRv amyloidosis diagnosis, mean (range), years	64.6 (33–82)	59.3 (26–78)	63.4 (26–82)
Method of confirming diagnosis^b^, *n* (%)			
Technetium scan (PYP)	20 (44.4)	4 (30.8)	24 (41.4)
Cardiac biopsy	5 (11.1)	1 (7.7)	6 (10.3)
Fat pad biopsy	1 (2.2)	5 (38.5)	6 (10.3)
Salivary gland biopsy	3 (6.7)	0	3 (5.2)
Sural nerve biopsy	0	3 (23.1)	3 (5.2)
Other biopsy	18 (40.0)	5 (38.5)	23 (39.7)
ATTRv amyloidosis treatment received concurrently with patisiran, *n* (%)			
Yes^c^	17 (37.8)	8 (61.5)	25 (43.1)
Tafamidis^d^	12 (26.7)	4 (30.8)	16 (27.6)
Diflunisal^e^	5 (11.1)	3 (23.1)	8 (13.8)
Doxycycline	4 (8.9)	3 (23.1)	7 (12.1)
Inotersen	1 (2.2)	1 (7.7)	2 (3.4)
Tauroursodeoxycholic acid	1 (2.2)	0	1 (1.7)
No	28 (62.2)	5 (38.5)	33 (56.9)
PND score, *n* (%)			
I: Preserved walking, sensory disturbances	26 (57.8)	7 (53.8)	33 (56.9)
II: Impaired walking, but can walk without stick/crutch	13 (28.9)	3 (23.1)	16 (27.6)
IIIA: Walk with one stick/crutch	3 (6.7)	2 (15.4)	5 (8.6)
IIIB: Walk with two sticks/crutches	3 (6.7)	1 (7.7)	4 (6.9)
Karnofsky Performance Status^f^, *n* (%)			
100%	1 (2.2)	1 (7.7)	2 (3.4)
90%	8 (17.8)	5 (38.5)	13 (22.4)
80%	21 (46.7)	4 (30.8)	25 (43.1)
70%	13 (28.9)	2 (15.4)	15 (25.9)
60%	2 (4.4)	1 (7.7)	3 (5.2)
NYHA class^g^, *n* (%)			
No HF	5 (11.1)	1 (7.7)	6 (10.3)
I: No symptoms with normal physical activity; normal functional status	12 (26.7)	4 (30.8)	16 (27.6)
II: Mild symptoms with normal physical activity; slight limitation of functional status	26 (57.8)	7 (53.8)	33 (56.9)
III: Moderate symptoms with less than normal physical activity; comfortable only at rest; marked limitation of functional status	2 (4.4)	1 (7.7)	3 (5.2)

^a^Age was computed as informed consent year minus year of birth.

^b^Some patients’ diagnosis was confirmed by > one method, so percentages do not sum to 100%.

^c^Patients could take > one medication, so percentages do not sum to 100%.

^d^Fifteen patients receiving tafamidis (93.8%) initiated patisiran treatment due to neuropathy progression.

^e^Six patients on (off-label) diflunisal (75.0%) initiated patisiran treatment due to neuropathy progression.

^f^Decreasing Karnofsky Performance Status indicates worsening performance status.

^g^Patients with NYHA Class 4 HF at baseline were excluded from the study.

ATTRv, variant transthyretin amyloidosis; HF, heart failure; NYHA, New York Heart Association; PND, polyneuropathy disability; PYP, technetium pyrophosphate scintigraphy.

Of the 58 patients treated, the majority (50 (86.2%)) were prescribed patisiran after experiencing disease progression. Among the 50 patients who initiated patisiran due to disease progression, 26 (52.0%) experienced progression due to neuropathy only; 20 (40.0%) had progression due to neuropathy and cardiac disease; and 4 (8.0%) had progression due to cardiac disease only. All four patients who initiated patisiran due to cardiac disease progression only had evidence of polyneuropathy at baseline (PND score ≥ 1), consistent with the study protocol and the prescribing information for patisiran. During the study (from patisiran initiation to end of study), 25 (43.1%) patients were treated with ≥ 1 ATTRv amyloidosis medication in addition to patisiran (Table 1), with 16 (27.6%) receiving tafamidis and eight (13.8%) receiving diflunisal.

Patients experienced a range of ambulatory dysfunctions at baseline, with 33 (56.9%), 16 (27.6%), five (8.6%), and four (6.9%) having a PND score of I, II, IIIA, and IIIB, respectively. At baseline, patients had evidence of impaired quality of life due to neuropathy, with a mean (SE) Norfolk QOL-DN of 28.4 (5.08). HF-related health status by mean (SE) KCCQ-OS was 63.97 (5.22). Impaired autonomic function, as shown by mean (SE) COMPASS-31 of 22.40 (3.09), was also observed. The safety population also showed evidence of cardiac dysfunction at baseline, with elevated mean (SE) NT-proBNP of 1239.67 (744.50) ng/L and 36 (62.1%) patients in NYHA Class II or III. Among the prospective cohort (*n* = 27), 10 (37.0%) patients had been hospitalized for HF. At baseline, 52 (89.7%) patients had HF, the majority of whom (45 (86.5%)) had PND scores of I or II; some patients with no HF (*n* = 6) still had neurologic involvement, with PND scores ranging between I and IIIb (Table S1). Patients with no HF tended to be younger than patients with HF (Table S1).

### Relevant surgical and medical history

A total of 24 (41.4%) patients had ≥ 1 relevant finding associated with ATTRv amyloidosis in their surgical history (Table 2). Ten (17.2%) patients had undergone a cardiac device therapeutic procedure, two (3.4%) had undergone aortic valve replacement, and four (6.9%) had undergone other cardiac procedures. In addition, patients underwent orthopedic procedures, often associated with amyloid deposition, in their musculoskeletal and/or soft tissues: six patients (10.3%) underwent joint procedures such as hip arthroplasty and rotator cuff repair, five (8.6%) underwent peripheral nerve procedures such as carpal tunnel decompression, and five (8.6%) underwent spine and spinal cord procedures such as spinal laminectomy and spinal operation (*n* ≤ 5 for each condition). Past medical histories indicated the presence of significant polyneuropathy, including autonomic and sensory/motor dysfunction (Table 2).

**Table 2.** Relevant surgical and medical history in patients with polyneuropathy (safety population).

	V122I variant (*n* = 45)	T60A variant (*n* = 13)	Total (*n* = 58)
Relevant surgical history, *n* (%)^a^			
At least one relevant surgical history finding^b^	17 (37.8)	7 (53.8)	24 (41.4)
Cardiac device therapeutic procedures^c^	7 (15.6)	3 (23.1)	10 (17.2)
Cardiac pacemaker insertion^d^	4 (8.9)	1 (7.7)	5 (8.6)
Implantable defibrillator insertion^d^	5 (11.1)	2 (15.4)	7 (12.1)
Aortic valve replacement	2 (4.4)	0	2 (3.4)
Cardiac therapeutic procedures NEC^e^	3 (6.7)	1 (7.7)	4 (6.9)
Joint therapeutic procedures^f^	3 (6.7)	3 (23.1)	6 (10.3)
Peripheral nerve therapeutic procedures	4 (8.9)	1 (7.7)	5 (8.6)
Carpal tunnel decompression	3 (6.7)	1 (7.7)	4 (6.9)
Spine and spinal cord therapeutic procedures^g^	5 (11.1)	0	5 (8.6)
Relevant medical history, *n* (%)^a^			
At least one relevant medical history finding^h^	28 (62.2)	7 (53.8)	35 (60.3)
Carpal tunnel syndrome	8 (17.8)	1 (7.7)	9 (15.5)
Neuropathy peripheral	4 (8.9)	0	4 (6.9)
Heart failures NEC	21 (46.7)	1 (7.7)	22 (37.9)
Cardiac failure congestive^d^	20 (44.4)	1 (7.7)	21 (36.2)
Cardiac disorders relevant to polyneuropathy^i^			
Atrial fibrillation	5 (11.1)	1 (7.7)	6 (10.3)
Postural orthostatic tachycardia syndrome	1 (2.2)	0	1 (1.7)
Orthostatic hypotension	0	1 (7.7)	1 (1.7)
Syncope^d^	6 (13.3)	0	6 (10.3)
Tachycardia	1 (2.2)	0	1 (1.7)

^a^Data reported based on the MedDRA High Level and Preferred Terms categorization.

^b^High Level terms of all relevant surgical history findings: bladder therapeutic procedures, cardiac device therapeutic procedures, cardiac therapeutic procedures NEC, cardiac valve therapeutic procedures, joint therapeutic procedures, limb therapeutic procedures, peripheral nerve therapeutic procedures, and spine and spinal cord therapeutic procedures.

^c^If a patient has more than 1 condition in a given High Level term, or Preferred Term, the patient is counted once for that High Level term, or Preferred Term, respectively.

^d^These data include medical history only collected in the prospective cohort (*n* = 27: V122I (*n* = 26); T60A (*n* = 1)).

^e^Atrial appendage closure, cardiac ablation, heart and lung transplant, heart transplant (*n* < 5 for each condition).

^f^Ankle operation, hip arthroplasty, joint injection, knee operation, ligament operation, meniscus operation, rotator cuff repair (*n* < 5 for each condition).

^g^Intervertebral disc operation, spinal laminectomy, spinal operation (*n* < 5 for each condition).

^h^High Level terms of all relevant medical history findings: autonomic nervous system disorders, cardiac conduction disorders, cardiac signs and symptoms NEC, cardiomyopathies, central nervous system hemorrhages and cerebrovascular accidents, cervical spinal cord and nerve root disorders, coronary artery disorders NEC, disturbances in consciousness NEC, heart failures NEC, ischemic coronary artery disorders, left ventricular failures, lumbar spinal cord and nerve root disorders, migraine headaches, mitral valvular disorders, mononeuropathies, myocardial disorders NEC, paresthesias and dysesthesias, peripheral neuropathies NEC, rate and rhythm disorders NEC, sensory abnormalities NEC, spinal cord and nerve root disorders NEC, supraventricular arrhythmias, transient cerebrovascular events, tricuspid valvular disorders, vascular hypotensive disorders, and ventricular arrhythmias and cardiac arrest.

^i^Cardiac disorders relevant to polyneuropathy include manifestations of cardiac autonomic neuropathy.

MedDRA, Medical Dictionary for Regulatory Activities; NEC, not elsewhere classified.

### Primary endpoint

**Figure 2.**
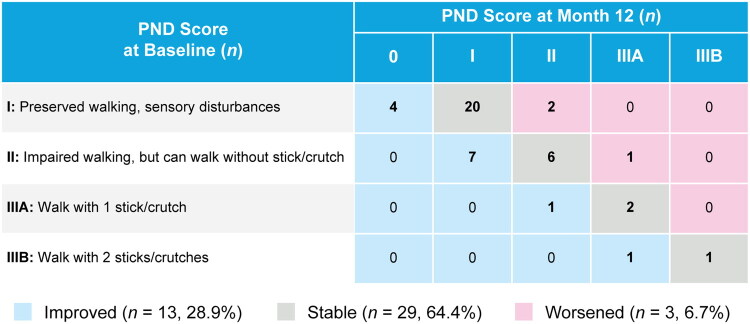
Overall PND score at Month 12 relative to first dose of patisiran (efficacy population, *n* = 45). PND, polyneuropathy disability.

In the efficacy population, 42/45 (93.3%) patients demonstrated stable or improved PND scores from baseline to Month 12 after patisiran treatment was initiated (Figure 2), of whom 29 (64.4%) patients had stable scores and 13 (28.9%) had improved scores (Figure 2). Of the three (6.7%) patients who worsened, all had diagnoses potentially contributing to polyneuropathy (*n* = 2 diabetes, and *n* = 1 Ehlers–Danlos syndrome [20]). The proportion of patients with stable or improved PND scores was consistent in patients receiving and not receiving concurrent TTR stabilizer treatment (data not shown).

### Exploratory endpoints

**Figure 3.**
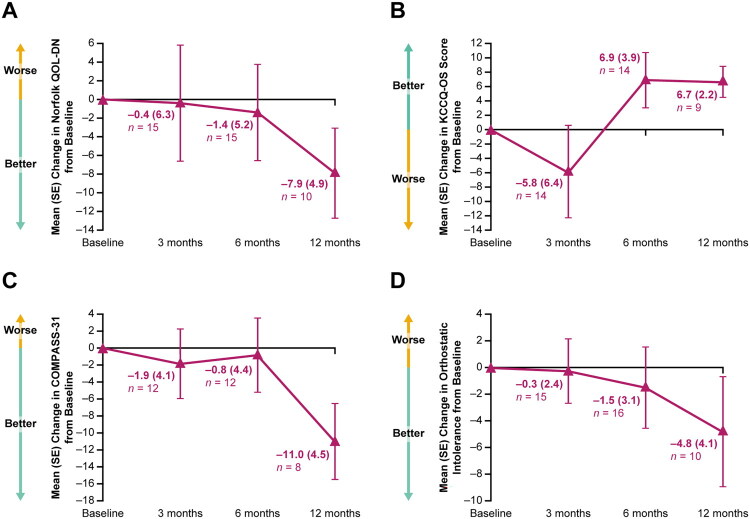
Mean (SE) change from baseline to Month 12 in (A) Norfolk QOL-DN total score, (B) KCCQ-OS total score, (C) COMPASS-31 total score, and (D) orthostatic intolerance (efficacy population, *n* = 45). Mean (SE) Norfolk QOL-DN score at baseline was 28.44 (5.08), with a range of –2.0 to 78.0; higher scores of Norfolk QOL-DN indicate worse quality of life (range: –4 to 136). Mean (SE) KCCQ-OS score at baseline was 63.97 (5.22); lower KCCQ-OS scores indicate worse health status (range 0–100). Mean (SE) COMPASS-31 score at baseline was 22.40 (3.09), with a range of 0.0–45.7. Mean (SE) orthostatic intolerance score at baseline was 9.38 (1.93), with a range of 0.0–32.0; higher scores of COMPASS-31 total (range: 0–100) and orthostatic intolerance (range 0–40) indicate worse autonomic symptoms. Orthostatic intolerance is a domain of COMPASS-31. COMPASS-31, Composite Autonomic Symptom Score-31; KCCQ-OS, Kansas City Cardiomyopathy Questionnaire – Overall Summary; Norfolk QOL-DN, Norfolk Quality of Life – Diabetic Neuropathy; SE, standard error.

Evaluable patients in the efficacy population demonstrated an improvement in Norfolk QOL-DN from baseline to Month 12 (mean (SE) change from baseline at Month 12, −7.9 (4.9), *n* = 10), with the trend for improvement evident as early as Month 3 (Figure 3(A)). Patients demonstrated an improvement in KCCQ-OS (mean (SE) change from baseline at Month 12, 6.7 (2.2), *n* = 9), starting at Month 6 of patisiran treatment (Figure 3(B)), and improvement in COMPASS-31 (mean (SE) change from baseline at Month 12, −11.0 (4.5), *n* = 8), starting at Month 3 of patisiran treatment (Figure 3(C)). Improvement was also observed in the orthostatic intolerance domain of COMPASS-31, with mean (SE) change from baseline at Month 12 of −4.8 (4.1) (*n* = 10); improvement was evident after 3 months (Figure 3(D)). Mean (SE) changes from baseline at Month 12 in the vasomotor (*n* = 10), secretomotor (*n* = 10), gastrointestinal (*n* = 9), bladder (*n* = 10), and pupillomotor (*n* = 8) domains of COMPASS-31 were −0.3 (0.4), −0.2 (0.8), −1.0 (1.1), 0.2 (0.5), and 0.0 (0.2), respectively.

**Figure 4.**
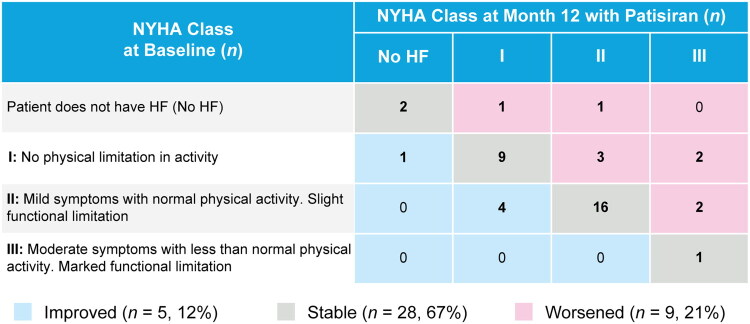
Change in NYHA classification from baseline to month 12 in patisiran-treated patients (safety population, *n* = 58). HF, heart failure; NYHA, New York Heart Association.

NT-proBNP change from baseline at Month 12 was only evaluable in six patients and remained stable (mean (SE) change from baseline to Month 12, 11.1 (19.6) ng/L). NYHA Class remained stable or improved in 33/42 (78.6%) evaluable patients at Month 12 (Figure 4).

### Safety

Safety was summarized for the prospective and ambispective cohorts (*n* = 42), with patients in the retrospective cohort excluded due to safety information only being collected prospectively. In total, for 25.2 patient years of exposure, adverse events (AEs) were reported in 13/42 (31.0%) patients with an exposure-adjusted incidence rate of 0.52 events per patient-year; the majority of AEs were mild or moderate in intensity (Table 3).

**Table 3.** Summary of selected safety events by decreasing incidence in prospective and ambispective patients (*n* = 42).

Safety summary^a^	Patients with event, *n* (%) (*n* = 42)	Exposure-adjusted incidence rate, rate/patient years (patient years = 25.2)
At least one AE	13 (31.0)	0.52
AEs occurring in > 1 patient		
Congestive cardiac failure	2 (4.8)	0.08
Infusion-related reaction	2 (4.8)	0.08
AE related to patisiran^b^	3 (7.1)	0.12
Any serious AE^c^	9 (21.4)	0.36
AE leading to study withdrawal	2 (4.8)	0.08
Death^d^	4 (9.5)	0.16

^a^Selected AEs included events occurring or worsening after the first dose of patisiran and included death, significant AEs that led to an intervention, marked laboratory abnormalities, overdose, pregnancy, and adverse drug reactions. Selected safety events with missing causality were considered related. Selected safety events with missing severity were considered severe.

^b^There were three drug-related treatment-emergent AEs, none of which led to study withdrawal.

^c^All serious and severe safety events were considered unrelated to use of patisiran.

^d^All four deaths were considered to be unrelated to patisiran: acute respiratory failure (*n* = 1), cardiac failure (*n* = 1), cardiogenic shock (*n* = 1), unknown (*n* = 1).

AE, adverse event.

There were four deaths during the 12-month study: acute respiratory failure (*n* = 1), cardiac failure (*n* = 1), cardiogenic shock (*n* = 1), and unknown (*n* = 1). None were considered related to treatment with patisiran. Two of the patients who died also withdrew from the study due to AEs, neither of which were considered related to patisiran. In one patient, study withdrawal was due to a severe AE of peripheral edema that was not recovered/resolved; this patient also experienced cardiac failure, resulting in their death. The other patient experienced severe concomitant AEs of acute respiratory failure and congestive cardiac failure, which resulted in study withdrawal and were the reported causes of death.

Eleven patients were hospitalized during the study; four of the hospitalizations were associated with congestive HF and three hospitalized patients subsequently died. All hospitalizations were considered unrelated to patisiran. AEs that were reported in more than one patient were congestive cardiac failure (*n* = 2, 4.8%) and infusion-related reactions (*n* = 2, 4.8%) (Table 3). There were no safety concerns with patisiran regarding hepatotoxicity, hematology parameters, or renal function.

## Discussion

Over the past 10 years, there have been significant advances in the development of treatments targeting the amyloidogenic cascade of ATTRv amyloidosis. Approved and available therapies include TTR stabilizers such as tafamidis and acoramidis (indicated for ATTR amyloidosis with cardiomyopathy) [21,22], as well as gene-silencing therapeutics that reduce expression of serum TTR by either RNAi (patisiran (indicated for polyneuropathy) and vutrisiran (indicated for polyneuropathy and cardiomyopathy)) [12,23–25] or antisense oligonucleotide-based mechanisms (inotersen and eplontersen (indicated for polyneuropathy)) [26]. However, patients with the V122I and T60A variants have generally been under-represented in clinical trials of patients with ATTRv amyloidosis with polyneuropathy [12,23,26], including the phase III APOLLO study of patisiran [12]. The current study was therefore designed to assess the efficacy and safety of patisiran in patients with these variants. We found that patisiran was able to stabilize or improve neurologic and cardiac endpoints in patients with V122I or T60A ATTRv amyloidosis. These data are consistent with those from pivotal studies of patisiran in patients with ATTRv amyloidosis with polyneuropathy (phase II open-label extension and APOLLO) or ATTR amyloidosis with cardiomyopathy (APOLLO-B) [12,27,28], and with real-world experience, which also shows beneficial effects of patisiran on neurologic and cardiac manifestations [29–31].

The V122I and T60A variants have been historically associated with predominant cardiomyopathy manifestations [7,8]. However, the present data add to the growing body of literature indicating that patients with these variants can also develop polyneuropathy. In this study, around one-third of patients had experienced both cardiac and neuropathic progression prior to patisiran initiation and nearly half experienced neuropathic progression only. The study also included six patients who had polyneuropathy and no HF. Just under half of patients had a PND score ≥ II and around two-thirds of patients were in NYHA Class ≥ II, with patients showing elevated NT-proBNP levels and high rates of HF at baseline. These results are in keeping with findings from the Transthyretin Amyloidosis Outcome Survey (THAOS) registry, which observed multisystem involvement regardless of TTR mutation [3]. A large biobank analysis found a significant association between the V122I variant and a polyneuropathy diagnosis, with a reported prevalence of polyneuropathy in V122I carriers of 2.1%, 9.0%, and 4.8% in the UK Biobank, Penn Medicine Biobank, and Million Veteran Program, respectively [6]. In addition, a single-center study found that over half of 36 patients with ATTRv amyloidosis and the V122I mutation had signs or symptoms attributable to polyneuropathy [32], and a study of 15 patients with the T60A mutation in Ireland found that neurologic manifestations pre-dated cardiac manifestations and were found in 93% of patients at diagnosis [9]. The V122I mutation was also identified in 14% of positive cases in a genetic screening study of patients in Sicily with clinical suspicion of ATTRv amyloidosis with polyneuropathy, further highlighting the importance of this variant in ATTRv amyloidosis with polyneuropathy globally [33]. This multisystem disease phenotype can be expected based on the pathophysiology of ATTRv amyloidosis, in which widespread deposition of amyloid has been reported [33–38] and the wide-ranging symptomatology of the disease underpins the need for treatments that impact the underlying cause.

Among evaluable patients, PND score was stabilized or improved in > 90% of those treated with patisiran. This is in contrast to the expected natural history of the disease in which rapid disease progression is typical, with a significantly faster progression rate *vs*. other peripheral neuropathies such as diabetic peripheral neuropathy [39]. For example, a single-center study found that median survival after diagnosis was 4.7 years in patients not receiving disease-modifying treatment [40], and stabilization of PND score over 18 months was observed in only 30% of the 77 placebo-treated patients in the APOLLO study [12]. Although our study had a relatively short duration (12 months), a global open-label extension study and a large, multicentered, Italian study corroborate the sustained clinical stability that patisiran provides for patients with ATTRv amyloidosis [41,42].

Whilt PND score is a frequently utilized assessment of polyneuropathy in patients with ATTRv amyloidosis, it can be less sensitive to small treatment effects and does not fully capture the multisystem involvement observed in these patients. As such, this study included a range of endpoints to capture the holistic impact of patisiran on patients with ATTRv amyloidosis who have these two variants, including measures of quality of life, overall health status, and autonomic and cardiac symptoms. We found that patisiran treatment led to improvement or stabilization of this broad group of endpoints compared with baseline. This is consistent with results seen in other patisiran clinical trials including APOLLO [12] and APOLLO-B [27], as well as the phase III HELIOS-A and HELIOS-B studies of the RNAi therapeutic vutrisiran in ATTRv amyloidosis with polyneuropathy and ATTR with cardiomyopathy, respectively [14,25], and a phase III trial of the antisense oligonucleotide inotersen in ATTRv amyloidosis with polyneuropathy [26]. Of note, patisiran acted rapidly in the current study, with positive effects on Norfolk QOL-DN and COMPASS-31 seen as early as 3 months after treatment initiation. Another real-world study of 15 patients with ATTRv amyloidosis reported a similar rapid effect of patisiran on 6-minute walk test distance within 9 months of treatment initiation, which was accompanied by stabilization of polyneuropathy, an increase in muscle mass, and a reduction in fat mass [43].

Patisiran was generally well tolerated in this small study of patients with V122I/T60A ATTRv amyloidosis with polyneuropathy, with a safety profile consistent with that from the larger, placebo-controlled APOLLO and APOLLO-B studies [12,44]. No deaths or cardiac hospitalizations were related to patisiran, and there were no concerns relating to hepatotoxicity, hematology parameters, or renal function.

One of the main limitations of this study is its small sample size and the fact that the study was not powered for statistical analysis. Although a larger population would help to demonstrate the consistency and generalizability of the findings, the observed results are consistent with previous trials [12]. In addition, the study lacked a placebo arm for comparison. However, the natural history of ATTRv amyloidosis is well defined and known to be characterized by progressive deterioration [33,45–47], whereas we found that patients receiving patisiran showed stabilization or improvement across a broad range of endpoints. It remains unclear whether disease progression of V122I or T60A is slower compared with other mutation types; establishing a control group would be necessary to address this question. In addition, inclusion of neurophysiologic measures and imaging as endpoints might have provided more information on disease progression. The study was relatively short, and a longer follow-up period would have yielded additional efficacy and safety data. Furthermore, around half of patients were receiving concomitant treatment(s) during the trial, which may have had an impact on the efficacy and safety findings. Finally, differences between the three patient cohorts in the study (retrospective, ambispective, and prospective) may have introduced variability across the patient population.

Patisiran demonstrated a consistent positive effect across multiple endpoints in patients with V122I/T60A ATTRv amyloidosis with polyneuropathy, with the potential to stabilize or improve manifestations of polyneuropathy with 12 months of treatment. Efficacy and safety results are consistent with those from the pivotal patisiran studies, further supporting the use of commercial patisiran in these patients. Patients with V122I/T60A ATTRv amyloidosis may often have multisystem disease with both cardiomyopathy and peripheral neuropathy, highlighting the importance of a treatment that demonstrates benefit across the multisystem manifestations of this disease.

## Supplementary Material

Supplement.docx

CONSORT 2010 Checklist Hussian Patisiran Phase 4.doc

## Data Availability

De-identified individual participant data that support these results will be made available through the website www.vivli.org, a secure-access environment, following an embargo, due to commercial restrictions, of 12 months after study completion and when the product and indication have been approved for no less than 12 months in the US and the EU. Access will be provided on reasonable request, contingent upon the approval of a research proposal and the execution of a data sharing agreement. Requests for access to data can be submitted *via* the website.

## Abbreviations

ATTRv: variant transthyretin
PND: polyneuropathy disability
