# Supplementary material for: Impact of patisiran on polyneuropathy of hereditary transthyretin amyloidosis in patients with a V122I or T60A variant: a phase IV multicenter study
Source: Ann Med. 2025 Sep 20;57(1):2537347. doi: 10.1080/07853890.2025.2537347 (PMC12451958; doi:10.1080/07853890.2025.2537347)
Supplement: Supplement.docx [file IANN_A_2537347_SM6811.docx]

# Supplementary appendix

## Supplementary methods

### Efficacy endpoints

- The primary endpoint was the effectiveness of patisiran on ambulatory status, measured by the proportion of patients with stable or improved polyneuropathy disability (PND) score at 12 months relative to baseline (first dose of patisiran).
- Exploratory endpoints included:
  - Norfolk Quality of Life – Diabetic Neuropathy questionnaire, a 35-item patient-reported outcomes measure that assesses six domains: physical function, large-fiber neuropathy, activities of daily living, small-fiber neuropathy, and autonomic neuropathy. Score range is –4–136, with higher scores indicating worse quality of life [1].
  - Kansas City Cardiomyopathy Questionnaire, a 23-item self-administered questionnaire that measures the patient’s perception of health status, and includes heart failure (HF) symptoms, impact on physical and social function, and how their HF impacts their quality of life within a 2-week recall period. Score range is 0–100, with lower scores indicating worse health status [2].
  - Composite Autonomic Symptom Score-31 questionnaire, which consists of 31 clinically selected questions evaluating the six autonomic symptoms (orthostatic intolerance, vasomotor, secretomotor, gastrointestinal, bladder, and pupillomotor). Score range is 0–100, with higher scores indicating more autonomic symptoms [3].
  - New York Heart Association class: clinical assessment of symptoms resulting from HF, ranging from Class 1 (no symptoms) to Class 4 (symptoms at rest); assessed at baseline and at Month 12.
  - N-terminal prohormone of brain natriuretic peptide: measure of cardiac stress, with higher values indicating a greater level of cardiac stress.

### Data collection

The PND score was assessed at baseline (time of first dose of patisiran) and thereafter at the time of routine clinical follow-up appointments (every 3, 6, and 12 months in the USA). Exploratory endpoints were assessed also at Months 3, 6, and 12. Missed or rescheduled visits did not lead to automatic discontinuation and were not considered to be a protocol deviation.

For both retrospective and ambispective patients, baseline data were abstracted from the medical chart for the most recent assessments prior to first dose of patisiran. For the prospective cohort, patients had to receive the first dose of patisiran within 45 days of consent or undergo baseline assessments again before study initiation. At the end of the study, patients in the prospective or mixed cohorts who withdrew or who discontinued from the study early (i.e. prior to Month 12) were encouraged to complete end-of-study assessments at the next routine care appointment.

### Institutional Review Boards

- WCG IRB
- UCLA IRB
- VA Greater Los Angeles Healthcare System Research Service IRB
- Northwestern University IRB
- UNMC IRB
- UCSD Human Research Protections Program
- Medical College of Wisconsin/Froedtert Hospital IRB
- Cook County Health IRB
- University of Iowa IRB
- HFHS IRB
- UC Davis IRB
- Advarra IRB
- Mayo Clinic IRB
- St. Luke’s University Health Network IRB
- Orlando Health IRB
- Lancaster General Hospital IRB
- St. Luke’s Health System IRB
- Baylor Scott & White Research IRB
- Lehigh Valley Health Network IRB

## Supplementary tables

**Supplementary Table 1.** Baseline age demographics by PND score and HF status.

| Baseline assessment | | | | Mean (standard deviation), median (minimum, maximum) | | |
| --- | --- | --- | --- | --- | --- | --- |
| All patients | | | | | | |
| PND score | New York Heart Association class | *N* | *N* (symptom onset age) | Age at enrollment | Age at diagnosis | Age at symptom onset |
| Total | Total | 58 | 57 | 65.1 (12.64), 68 (30, 83) | 63.4 (12.62), 66 (26, 82) | 60.7 (14.60), 64 (18, 82) |
| Total | No HF | 6 | 6 | 57.5 (15.06), 51 (44, 78) | 56.5 (15.90), 50 (42, 78) | 51.8 (18.78), 49 (25, 77) |
| Total | HF | 52 | 51 | 65.9 (12.20), 68 (30, 83) | 64.2 (12.13), 67 (26, 82) | 61.7 (13.90), 64 (18, 82) |
| I | Total | 33 | 32 | 64.2 (11.58), 67 (30, 80) | 62.4 (11.81), 66 (26, 79) | 59.2 (13.96), 63 (18, 75) |
| I | No HF | 4 | 4 | 55.8 (13.15), 51 (46, 75) | 54.8 (13.89), 50 (44, 75) | 48.0 (18.02), 49 (25, 69) |
| I | HF | 29 | 28 | 65.4 (11.09), 67 (30, 80) | 63.4 (11.37), 66 (26, 79) | 60.8 (12.90), 64 (18, 75) |
| II | Total | 16 | 16 | 65.6 (14.17), 68 (33, 83) | 64.1 (13.76), 67 (33, 80) | 62.3 (15.17), 65 (25, 78) |
| II | HF | 16 | 16 | 65.6 (14.17), 68 (33, 83) | 64.1 (13.76), 67 (33, 80) | 62.3 (15.17), 65 (25, 78) |
| IIIA | Total | 5 | 5 | 69.0 (13.78), 76 (50, 82) | 68.8 (13.66), 75 (50, 82) | 64.6 (18.80), 75 (40, 82) |
| IIIA | No HF | 1 | 1 | 78.0, 78  (78, 78) | 78.0, 78 (78, 78) | 77.0, 77 (77, 77) |
| IIIA | HF | 4 | 4 | 66.8 (14.82), 68 (50, 82) | 66.5 (14.62), 67 (50, 82) | 61.5 (20.17), 62 (40, 82) |
| IIIB | Total | 4 | 4 | 64.8 (17.73), 68 (44, 80) | 62.3 (16.74), 66 (42, 76) | 61.5 (16.78), 64 (42, 76) |
| IIIB | No HF | 1 | 1 | 44.0, 44 (44, 44) | 42.0, 42 (42, 42) | 42.0, 42 (42, 42) |
| IIIB | HF | 3 | 3 | 71.7 (13.58), 79 (56, 80) | 69.0 (12.12), 76 (55, 76) | 68.0 (13.00), 75 (53, 76) |
| V122I patients | | | | | | |
| Total | Total | 45 | 44 | 66.1 (12.81), 70 (33, 83) | 64.6 (12.57), 69 (33, 82) | 61.8 (14.61), 65 (25, 82) |
| Total | No HF | 5 | 5 | 53.4 (12.54), 49 (44, 75) | 52.2 (13.31), 48 (42, 75) | 46.8 (15.83), 48 (25, 69) |
| Total | HF | 40 | 39 | 67.7 (12.07), 72 (33, 83) | 66.2 (11.74), 70 (33, 82) | 63.7 (13.48), 66 (25, 82) |
| I | Total | 26 | 25 | 65.3 (10.95), 68 (40, 80) | 63.8 (10.81), 67 (38, 79) | 60.5 (12.86), 63 (25, 75) |
| I | No HF | 4 | 4 | 55.8 (13.15), 51 (46, 75) | 54.8 (13.89), 50 (44, 75) | 48.0 (18.02), 49 (25, 69) |
| I | HF | 22 | 21 | 67.0 (9.89), 70 (40, 80) | 65.4 (9.65), 69 (38, 79) | 62.9 (10.60), 64 (33, 75) |
| II | Total | 13 | 13 | 66.1 (15.69), 72 (33, 83) | 64.5 (15.26), 71 (33, 80) | 62.8 (16.66), 66 (25, 78) |
| II | HF | 13 | 13 | 66.1 (15.69), 72 (33, 83) | 64.5 (15.26), 71 (33, 80) | 62.8 (16.66), 66 (25, 78) |
| IIIA | Total | 3 | 3 | 72.3 (11.93), 76 (59, 82) | 72.0 (11.79), 75 (59, 82) | 65.7 (22.50), 75 (40, 82) |
| IIIA | HF | 3 | 3 | 72.3 (11.93), 76 (59, 82) | 72.0 (11.79), 75 (59, 82) | 65.7 (22.50), 75 (40, 82) |
| IIIB | Total | 3 | 3 | 67.7 (20.50), 79 (44, 80) | 64.7 (19.63), 76 (42, 76) | 64.3 (19.35), 75 (42, 76) |
| IIIB | No HF | 1 | 1 | 44.0, 44 (44, 44) | 42.0, 42 (42, 42) | 42.0, 42 (42, 42) |
| IIIB | HF | 2 | 2 | 79.5 (0.71), 80 (79, 80) | 76.0 (0.00), 76 (76, 76) | 75.5 (0.71), 76 (75, 76) |

HF, heart failure; PND, polyneuropathy disability.

## Supplementary references

[1] Vinik EJ, Vinik AI, Paulson JF, et al. Norfolk QOL-DN: validation of a patient reported outcome measure in transthyretin familial amyloid polyneuropathy. J Peripher Nerv Syst. 2014;19(2):104–114.

[2] Green CP, Porter CB, Bresnahan DR, et al. Development and evaluation of the Kansas City Cardiomyopathy Questionnaire: a new health status measure for heart failure. J Am Coll Cardiol. 2000;35(5):1245–1255.

[3] Sletten DM, Suarez GA, Low PA, et al. COMPASS 31: a refined and abbreviated Composite Autonomic Symptom Score. Mayo Clin Proc. 2012;87(12):1196–1201.
